# Information transfer: what do decision makers want and need from researchers?

**DOI:** 10.1186/1748-5908-2-20

**Published:** 2007-07-03

**Authors:** Maureen Dobbins, Peter Rosenbaum, Nancy Plews, Mary Law, Adam Fysh

**Affiliations:** 1 School of Nursing, Faculty of Health Sciences, McMaster University, 1200 Main Street West, 3N25G, Hamilton, ON, L8N 3Z5, Canada; 2 CanChild Centre for Childhood Disability Research, McMaster University, IAHS Building, Room 408, 1400 Main Street West, Hamilton, ON, L8S 1C7, Canada

## Abstract

**Purpose:**

The purpose of this study was to undertake a systematic assessment of the need for research-based information by decision-makers working in community-based organizations. It is part of a more comprehensive knowledge transfer and exchange strategy that seeks to understand both the content required and the format/methods by which such information should be presented.

**Methods:**

This was a cross-sectional telephone survey. Questions covered current practices, research use, and demographic information, as well as preferences for receiving research information. Three types of organizations participated: Children's Treatment Centres of Ontario (CTCs); Ontario Community Care Access Centres (CCACs); and District Health Councils (DHCs). The analysis used descriptive statistics and analyses of variance (ANOVA) to describe and explore variations across organizations.

**Results:**

The participation rate was 70%. The highest perception of barriers to the use of research information was reported by the CCAC respondents, followed by CTCs and DHCs. The CTCs and DHCs reported greater use of research evidence in planning decisions as compared to the CCACs. Four sources of information transfer were consistently identified. These were websites, health-related research journals, electronic mail, and conferences and workshops. Preferred formats for receiving information were executive summaries, abstracts, and original articles.

**Conclusion:**

There were a number of similarities across organization type with respect to perceived barriers to research transfer, as well as the types of activities the organizations engaged in to promote research use in decision-making. These findings support the importance of developing interactive, collaborative knowledge transfer strategies, as well as the need to foster relationships with health care decision-makers, practitioners and policymakers.

## Background

### Knowledge translation and exchange

In recent years the terms knowledge transfer and exchange, evidence-based decision-making, and evidence-informed health policy have become commonly used, but with little consensus on their definition, how they occur, or can be promoted [1,2]. Furthermore, significant resources and time are invested in the production of research evidence that, if effectively transferred, could be used to inform policy and practice decisions and subsequently improve patient and population health outcomes [3]. In October 1994, the Prime Minister of Canada launched the National Forum on Health to involve and inform Canadians and to advise the federal government on innovative ways to improve the health system and the health of Canada's people. A key recommendation arising from the National Forum on Health [4] was the development of an evidence-informed health care system in Canada where policies and clinical decisions are influenced by high quality research knowledge. As a result, considerable research inquiry has focused on understanding the processes of evidence-informed decision-making, as well as on how to facilitate it.

In order to move toward an evidence-informed health care system, significant environmental changes are required. At a minimum, researchers must become more effective communicators of their research findings, gain a better appreciation of the context in which decision-makers function, and build more collaborative relationships with policy-makers, decision-makers and practitioners [3,5,6]. In addition, policy-makers, decision-makers and practitioners must become more receptive to the inclusion of the best available research evidence in the decision-making process, and be willing to collaborate with researchers to ensure that relevant and applicable research is conducted [7-9].

The knowledge transfer field in health care, while relatively young compared to other empirical fields, has evolved significantly in the past 40 years. It has identified barriers and facilitators of knowledge transfer, determined the effectiveness of dissemination strategies, explored decision-making processes and organizational capacity for change, and evaluated collaborative efforts to bring producers and users of research evidence together to develop, implement and interpret research evidence. Consistent barriers across all settings include lack of available time, lack of access to current research literature, limited critical appraisal skills, excessive literature to review, work environments that do not support research transfer and uptake, lack of decision-making authority to implement research results, organizational decision-making processes that are not conducive to research transfer and uptake, resistance to change, and limited resources for implementation [10-17].

While the process is complex and constantly changing, several generalizations about knowledge transfer and exchange (KTE) can be made. Traditional passive strategies used alone are relatively ineffective [18,19]. Strategies that are more interactive and involve face-to-face contact show promising results with a variety of target populations [12,20-23]. Involvement of decision-makers in the research process has been shown to be associated with higher degrees of uptake [24]. When the results or 'messages' of research results are tailored to the specific needs of decision-makers then perceived uptake is higher [3,25,26]. An emerging hypothesis is that a combination of strategies, resulting in an interactive, multi-component KTE program that reinforces relationships between research producers and users, and reaches potential users on multiple levels, may be most effective in achieving an evidence-informed health care system [27].

The CanChild Centre for Childhood Disability Research at McMaster University, funded since 1989 by the Ontario Ministry of Health and Long-Term Care, implements interactive, multi-component interventions that reinforce relationships between research producers and potential users. Two main goals of CanChild are to provide effective leadership and innovation in childhood disability research and information transfer, and to impact on knowledge, practice, services, and policy in childhood disability through programmatic research and research transfer [28]. CanChild accomplishes these goals by working collaboratively with community agencies, including all members of the Ontario Children's Treatment Centres (CTCs) and the Ontario Community Care Access Centres (CCACs), and to a modest extent the District Health Councils of Ontario.

Since its inception, CanChild has worked in partnership with all of the CTCs in Ontario. The CTCs provide developmental therapies and family-based services to children with a variety of developmental disabilities and their families [29]. More recently, CanChild has started working collaboratively with the CCACs of Ontario, which have become increasingly involved in the provision of services to children with disabilities. CCACs offer an access point to Ontario's long-term care system by: assessing and arranging for visiting health and professional services in people's homes; assessing, authorizing, and arranging for the provision of school health support services for children; providing information and referrals to the public about other community agencies and services available to them; and coordinating services such as nursing, physiotherapy, occupational therapy, speech-language therapy, dietician services, social work, personal support and homemaking [30]. The District Health Councils provide advice to the Ontario Ministry of Health and Long-Term Care on health needs and other health matters in their geographic areas. They also play an important role in the provision of health care information in their communities, as well as promote the integration of health services and identify health planning needs [31].

All three types of organizations were approached to participate in this study to explore different decision-makers' perspectives on knowledge translation. These organizations were chosen because they are all involved in developing services for children with disabilities, but have different relationships with CanChild. For example, at the time of this study there existed a longstanding partnership between the CTCs and CanChild, including a focused program of KTE between the two organizations. While CanChild had a working relationship with the CCACs, the relationship was in its infancy, and at the time of this study there had been fewer opportunities to promote the transfer and uptake of research information by CCACs. In addition, at the time of this study there was no formal, established relationship between the DHCs and CanChild. It was expected that variations in the respective missions and existing relationships with CanChild would provide interesting observations related to the uptake of research evidence in decision-making.

Among the most consistent issue identified by those working in the childhood disability field is the need for information that is of high quality, synthesized, easy to use, and easy to access [32]. In its attempt to address these information needs, CanChild reviewed the KTE literature to assist in the development of a KTE program that would facilitate relationships between research producers and users, and promote evidence-informed decision-making and practice [3,5,33-36]. Their review of the literature and interaction with the CTCs led to the adoption of these guiding principles. The dissemination source must be perceived as competent, credible, and trustworthy. The content must be perceived as relevant, usable, methodologically sound, and comprehensive to users. The medium must be accessible, user-friendly, and clearly understandable. Finally, the intended user must perceive the relevance of the materials to their own needs, and understand it in the context of their work.

These principles guided the development of the research dissemination program, which aims to enhance the incorporation of CanChild's and others' research evidence into policy and program decision-making [37]. A major objective is to facilitate the transfer and uptake of this research evidence specifically by CTCs and CCACs. The program is centered on the "Keeping Current" materials. These are brief 'bottom-line' systematic reviews of issues that have been assessed as being important 'hot topics' in the childhood disability field. They are written in plain language, are three to five pages in length, and can be read easily and quickly. Through Impact Surveys carried out by CanChild it has been shown that the 'Keeping Current' format is one that decision-makers and clinicians appreciate, and it has influenced thinking about issues and use of information [32]. While some evaluation has been conducted on this program, CanChild investigators have never systematically studied what information, in what formats, people want and need in their roles as clinicians, managers, directors, and CEOs. It is timely to evaluate the impact of such a program so that knowledge gained from this strategy can be used to enhance CanChild's program as well as inform KT strategies for other research-producing organizations.

It is understood that research uptake varies significantly across decision-making levels (*i.e*., CEOs, directors, managers, clinicians) Therefore, in this study it was recognized that responses would vary not only across the three organizational types, but also among the different decision-maker levels. For example, CEOs and senior managers/directors could utilize research evidence in decisions related to broad organizational policies concerning service provision or in recommendations for provincial health policies. Middle managers could use research evidence to inform decisions related to program planning. Clinicians could use research evidence to inform clinical practice, and senior health planners could use research evidence to inform recommendations for local and provincial resource allocation and service provision. Regardless of the decision type, it was expected that research evidence would be used by all participants in some way to make decisions during the study.

The purpose of this study was to undertake a systematic assessment of the need for research-based information by decision-makers working in community-based organizations. It is part of a more comprehensive knowledge transfer and exchange strategy that seeks to understand both the content required and the format/methods by which such information should be presented.

## Methods

### Design

The design was a cross-sectional telephone survey comprised of 65 questions (available from the first author upon request) that took approximately 20 to 25 minutes to complete.

### Respondents

Decision-makers at any level within the organization, ranging from CEOs to front-line clinicians (*i.e*., speech therapists, physiotherapists, occupational therapists) were invited to participate in the study. Senior health planners who worked only at DHCs were also recruited.

### Content of the survey

The survey asked questions about current practices, research use, and demographic information, as well as preferences for receiving research information. The survey was divided into five sections: 1) questions that sought to understand the current practices and culture of the organization were asked first, in order to provide the investigators with a more complete picture of the attitudes and motives of the respondents; 2) questions about access to research evidence, use by the individual, perceived barriers to the access, and use of research evidence; 3) questions about which formats and styles of research information were preferred, and the perceived use of research by the organization, as well as the field of health providers as a whole; 4) questions about possible mitigating factors in transferring information from researchers to service providers (*e.g*. established relationship between research producers and users), and whether the current circumstances of that transfer were satisfactory; and 5) questions about current practices, culture, barriers and mitigating factors, and the demographic structure of the organization and region.

### Scoring and data analysis

A variety of Likert scales, all consisting of five points, were used in this survey. Possible responses for questions 11 to 16 included 'Not an issue', 'a minor issue', 'a moderate issue', 'a serious issue', and 'a very serious issue'. For questions 36 to 41, the following response options were available: 'definitely won't', 'probably won't', 'may', 'probably will', and 'definitely will'. For questions 42 to 44 response options included: 'excellent', 'good', 'moderate', 'fair', 'poor'. Finally, for questions 46 to 56 responses included: 'strongly agree', 'moderately agree', 'neither agree or disagree', 'moderately disagree', 'strongly disagree'. Prior to analysis all of the scores were transformed to be consistent in direction, which meant scores ranged from one to five, with higher scores representing more positive perceptions.

Rather than relying exclusively on scores of individual items, groups of related items were scored and prorated according to the number of items in the group. Thus, for example, an 'integrated barrier score' was derived from items 11–16 of the questionnaire, grouped into scale scores based on a three-point rating which combined ratings that appeared similar. For example 'not an issue' and 'minor issue' were scored as 'generally not an issue', 'moderate issue' stayed as is, and 'a serious issue' and 'a very serious issue' were scored as 'generally a very serious issue'. After adjusting the data to reflect this reclassification, the average scores for each of items 11–16 were summed and divided by the total number of items (n = 6) to derive an 'integrated barrier score'. The same procedure was used to derive a 'research culture receptivity score' for items 45–50 of the questionnaire.

The analysis used descriptive statistics to report the patterns observed with the survey materials. Because item non-response varied, there are differences in the total number of participants for which data is available for different items. To explore variations across the three types of organizations involved in this study, analyses of variance (ANOVAs) were used, applying Tukey's multiple comparison test for post-hoc analyses where the ANOVA was significant at p < 0.05. Given the exploratory nature of this study, p < 0.05 was used so as to be more inclusive of potentially important variables.

### Administration of the survey

Respondents received an initial phone call to schedule a time to conduct the survey. The interviews were performed and data collected between January and April 2002. While research use tends to be overestimated by health care professionals when self-reported measures are used rather than a combination of direct observation and self-report [38], a self-reported telephone administered survey was adapted from previously published research [18,39] for this study. Two methods were used to reduce respondent overestimation of research use: respondents were assured their responses would be kept confidential and anonymous, and they were asked to give specific examples of research use. Previous research has demonstrated less overestimation of research use among public health professionals when concrete examples of research use are sought [39]. Furthermore, given that this was the first study of its kind to be conducted with this sample, it was expected that direct observation of individuals or teams would severely reduce participation in the study.

### Ethics

Ethics approval was sought and obtained from the McMaster University Research Ethics Board.

## Results

Overall the participation rate was moderate, with 92 of 131 potential respondents (70%) completing the survey. Table 1 provides a summary of participant characteristics from each organization, as well as type of decision-maker. The respondents varied in age, with the majority in the 40 to 59 year age group. The educational background of the respondents included a bachelor's degree n = 30 (32.6%); a master's degree n = 53 (57.6%); doctorate n = 1 (1.1%); and MBA n = 8 (8.7%). Respondents had been in their current roles for a mean of 5.5 (± 4.3) years, and in the field of childhood disability for a mean of 9.1 (± 9.3) years.

**Table 1:** Characteristics of participants

**Variable**	**CTCs N (%)**	**CCACs N (%)**	**DHS N (%)**	**Total**
Total Participation	28 (72)	38 (62)	26 (79)	91 (70)
Position				
CEO	12	14	9	35 (36)
Director/Manager	11	23	6	40 (38)
Senior Health Planner			11	11 (12)
Clinician	5	1	0	6 (6)

### Evidence of the validity of the questionnaire

Responses to four survey questions provide evidence of construct (discriminant) validity of the approach used in this survey. In response to the question: "With respect to its ease of use, how would you rate the quality of research information you have received recently?" there was a significant difference across the three organizations in favour of the CTCs. The overall mean score was 3.82 with mean scale scores of 4.11 for CTCs, 3.88 for DHCs, and 3.57 for the CCACs (p < 0.01). This finding was taken to reflect the active and collaborative relationship between CanChild and the CTCs that resulted in relevant and timely research being disseminated to them. In response to the statement: "My organization routinely actively looks for research information before making decisions", the overall mean score was 3.83, with mean DHC responses averaging 4.54, the CTCs 3.81, and the CCACs 3.35 (p < 0.01). This finding is thought to reflect the research nature of the DHCs and the 'clinical' focus of the other programs, in which research information informs, but is not essential to all decisions taken at the program planning and clinician level. Similarly, in response to the statement: "My organization provides ongoing training in research methods and critical appraisal to staff", the overall mean value was 2.75, with the DHC respondents reporting a mean of 3.50, the CTCs a mean of 2.63, and the CCACs a mean of 2.31 (p < 0.01). Finally, in response to the statement: "Overall, my organization provides adequate resources (financial or personnel) to implement decisions that are based on scientific evidence" the overall mean value was 3.62, and there was once again a gradient in favour of the DHC respondents (mean 4.04), compared with the CTCs (mean 3.52) and the CCACs (mean 3.43) (p = 0.04).

### Barriers to research transfer

Table 2 reports the relative importance of the barriers to research transfer, according to organization type and position. The only item where a statistically significant difference between organization type was observed for the question: 'to what degree is resistance to change at your organization a barrier to accessing and using research information in decision-making?' (p = 0.003). For this item the CCACs reported a significantly greater barrier with respect to resistance than either the CTCs or DHCs. When responses were analyzed by position and organization type, no statistically significant differences were observed for any of the barrier items.

**Table 2:** Barriers to using research in decision making

	Mean	To what degree is lack of time a barrier to you in the access and use of research information for decision making?	To what degree are the organization's limited financial resources a barrier to you in the access and use of research information for decision making?	To what degree is the availability of relevant research information a barrier to you in the access and use of research information for decision making?	To what degree is your limited training or experience in evaluating the quality of research material a barrier to you in the access and use of research information for decision making?	To what degree is resistance to change at your organization a barrier to you in the access and use of research information for decision making?	To what degree is availability of research information a barrier to you in the access and use of research information for decision making?
What kind of office is it?	N	91	91	90	91	90	91
CTC		3.5	2.5	2.5	2.5	1.6	1.9
CCAC		3.6	2.6	3.0	2.6	2.2	2.3
DHC		3.4	2.2	2.8	2.1	1.6	1.9
Overall mean (sd)		3.5 (1.0)	2.4 (1.1)	2.8 (1.1)	2.4 (1.0)	1.8 (0.8)***	2.0 (0.9)
Role							
Executive director		3.4	2.7	2.9	2.5	1.9	2.1
Director		3.8	2.4	2.6	2.2	2.2	2.1
Manager		3.6	2.7	2.8	2.7	1.5	2.3
Senior health planner		3.5	2.1	3.0	2.4	1.7	2.1
Clinician		3.5	1.9	2.6	2.3	1.8	1.7
Overall mean (sd)		3.5 (0.9)	2.5(1,1)	2.8(1.1)	2.4(1.0)	1.9(0.8)	2.1(0.9)

*p < 0.05, **p < 0.01, *** p < 0.001; Scale Points and anchors (1 = Strongly disagree, 2 = moderately disagree, 3 = neither agree nor disagree, 4 = moderately agree, 5 = strongly agree)

Data on the barrier items 11 to 16 were then aggregated and analyzed to determine if there were differences overall of perceptions of barriers between the three organization types. The mean overall barrier score on a three-point scale was 1.72 (higher scores report perception of greater barriers). Baseline characteristics such as age, type of organization being surveyed and respondent's highest level of education were not significantly associated with perceptions about barriers. There were statistically significant variations across organization type with the CCACs reporting the highest perception of barriers to the use of research information with a score of 1.88, while the CTCs reported a score of 1.65, and the DHCs a score of 1.59 (p < 0.006).

### Organizational characteristics

The data were also analyzed to explore associations between organizational characteristics and research use by organization type and position. The results are presented in Table 3. For all but one of the characteristics (mechanisms exist that facilitate the transfer of information), there were statistically significant differences across the three organization types. DHCs scored significantly higher on all items with the exception of impact of regulation and legislation where they scored lowest. When the data were analyzed by position there were statistically significant differences observed for two items: 'the organization provided ongoing training in research methods' (p = 0.03); and 'the organization makes decisions in collaboration with other health organizations' (p = 0.005). A third characteristic, 'the organization routinely looks for research information before making decisions', approached statistical significance (p = 0.053). For these characteristics, senior health planners' perceptions varied significantly from participants in other positions. For example, senior health planners perceived the organization provided significantly more training (3.63) than did directors (3.06) and managers (2.07). Senior health planners also perceived the organization to collaborate significantly more with other health organizations for decision-making (4.9), compared to directors (4.44) and clinicians (3.46).

**Table 3:** Perceptions of characteristics of the organization

	Mean	My organization routinely, actively looks for research information before making decisions	My organization provides ongoing training in research methods and critical appraisal to staff.	Mechanisms exist in my organization that facilitate the transfer of new information into the organization.	Overall, my organization provides adequate resources (financial or personnel) to implement decisions that are based on scientific evidence.	Regulations and legislation greatly impact on the decisions my organization makes about programs. (Provincial and/or local)	Most program decisions made at my organization are made in collaboration with other local health institutions or community agencies.
N		91	89	89	87	88	88
What kind of office is it?							
CTC		3.8	2.6	4.21	3.5	4.1	3.5
CCAC		3.4	2.3	4.2	3.4	4.9	4.1
DHC		4.5	3.5	4.3	4.0	4.0	4.9
Overall mean (sd)		3.8***(1.0)	2.8***(1.3)	4.2(0.8)	3.6*(0.9)	4.4***(0.9)	4.1***(1.0)
Role							
Executive Director		3.9	2.7	4.2	3.4	4.5	4.2
Director		4.0	3.1	4.4	3.7	4.4	4.4
Manager		3.3	2.1	4.3	3.4	4.9	3.8
Senior Health Planner		4.5	3.6	4.0	4.1	3.9	4.9
Clinician		3.8	2.5	4.0	3.9	4.2	3.5
Overall mean (sd)		3.8(1.0)	2.8*(1.3)	4.2(0.8)	3.6(0.9)	4.4(0.9)	4.1**(1.0)

*p < 0.05, **p < 0.01, ***p < 0.001; Scale Points and anchors (1 = Strongly disagree, 2 = moderately disagree, 3 = neither agree nor disagree, 4 = moderately agree, 5 = strongly agree)

Data on these organizational characteristics were then aggregated and analyzed to determine if there were differences of overall perceptions of organizational characteristics across the three organization types. These data are presented in Table 4. The mean overall organizational culture score on a five-point scale was 3.8 (higher scores are associated with a 'better' research culture). There was a statistically significant difference across organization type with DHCs, not surprisingly given their predominant focus on research activities, scoring the highest and the two service program organizations, CCACs and CTCs, reporting similar lower scores, CCACs and CTCs (p < 0.01).

**Table 4:** Mean scores of integrated culture scores by organization

What kind of office is it?	Mean	N
CTC	3.6	27
CCAC	3.7	36
DHC	4.2	26
Overall	3.8**	89

*p < 0.05, **p < 0.01, ***p < 0.001 Scale Points and anchors (1 = Strongly disagree, 2 = moderately disagree, 3 = neither agree nor disagree, 4 = moderately agree, 5 = strongly agree)

### Decision-makers' perceptions of knowledge translation strategies

People were asked two sets of questions about their preferred ways of receiving research information. The first set asked about each of several ways individually; the second asked people to rank their preferences. Table 5 reports the results of analyzing respondents' feelings about several possible methods for receiving information by organization and position. Those for which more than 90% of participants responded yes included conferences/workshops, short summaries, and colleagues and professional journals, while listservs were least preferred. There were no statistically significant differences in preferred methods for receiving research information according to position, and only one statically significant difference was observed at the organizational level: DHCs (46% saying yes) preferred listservs considerably more than CTCs (16.7%) and CCACs (5.4%). When the ranked methods of receipt of research information were assessed and weighted for organization type and position, four knowledge transfer methods stood out as being preferred most by the participants: websites, health-related research journals, electronic mail, and conferences/workshops.

**Table 5:** Preferred methods for receiving research information (% saying yes) (n = 91)

**Position**	**Website**	**Email**	**Newsletter**	**List Serv**	**Media Release**	**Health Related Journals**	**Professional Journals**	**Colleagues**	**Conferences Workshop**	**Short Summaries**
% of sample-preferring method	96.7	79.3	80.4	18.7	63.0	88.0	91.3	91.3	96.7	95.7
Executive Director	89	74.3	77.1	14.3	71.4	85.7	100	85.7	97.1	97.1
Director	79	83.3	88.9	16.7	50	94.4	88.9	100	100	100
Manager	86	78.6	92.9	7.1	57.1	92.9	100	85.7	92.9	71.4
SHP	91	90.1	63.6	36.4	90.1	81.8	81.8	90.1	90.1	91.1
Clinician	77	76.9	76.9	30.8	38.5	84.6	84.6	100	100	100
F	0.4	0.4	1.1	1.5	2.4	0.3	0.9	1.2	0.7	1.4
What kind of office is it?										
CTC	78.6	67.9	75	16.7	46.4	82.1	89.3	96.4	100	100
CCAC	86.5	78.4	89.2	5.4	67.6	91.9	91.9	89.2	94.6	94.6
DHC	88.5	92.3	73.1	46.2	73.1	88.5	92.3	88.5	96.2	92.3
F	1.4	2.5	1.8	19.5***	2.5	1.1	0.0	0.7	0.7	1.0

*SHP = Senior Health Planner; *p < 0.05, **p < 0.01, ***p < 0.001;

Questions about the preferred formats for receiving information showed that people's first choices were for executive summaries (53.2%), abstracts (29.3%) and original articles (17.4%). Second choices were for abstracts (45.7%), original articles (28.2%) and executive summaries (26.1%).

### Assessment of perceived impact of research information on decision-making

Four criteria were assessed to explore the use of research information in program decision-making. The results are presented in Table 6. There was a statistically significant difference between organization type for only one outcome: 'Research information provided justification for service/program decisions made by my organization', with DHCs rating this item significantly higher than CTCs and the CCACs (p < 0.035). When the data were analyzed by position, there was a statistically significant difference only for: 'Research information has resulted in a decision by your organization to conduct program evaluations'. In this instance, directors were much more likely to perceive that research information resulted in more program evaluations compared to senior health planners and clinicians (p < 0.004).

**Table 6:** Perception of use of research information in program planning decisions

What kind of office is it?	Mean	Research information has influenced program planning decisions at my organization	Research information has provided justification for service/program decisions made by my organization	Research information has resulted in a decision by your organization to conduct program evaluations	Research information has resulted in decisions to provide staff development training in your organization
N		91	89	89	91
What kind of office is it?					
CTC		4.1	4.2	3.0	4.0
CCAC		3.8	3.9	3.2	3.8
DHC		4.3	4.4	2.8	3.7
Overall mean (sd)		4.0(0.7)	4.1*(0.8)	3.0(1.3)	3.8(1.0)
Role					
Executive Director		4.2	4.2	2.9	3.9
Director		3.9	3.9	4.0	3.9
Manager		3.7	3.9	2.9	3.7
Senior Health Planner		4.4	4.6	2.3	3.7
Clinician		3.9	3.9	2.7	3.7
Overall mean (sd)		4.0(0.7)	4.1(0.8)	3.0**(1.3)	3.8(1.0)

*p < 0.05; **p < 0.01; ***p < 0.001 Scale Points and anchors (1 = Strongly disagree, 2 = moderately disagree, 3 = neither agree nor disagree, 4 = moderately agree, 5 = strongly agree)

## Discussion

The primary aim of this study was to identify information needs and preferences for research information, perceived barriers to using research evidence, and perceptions of use of research evidence among three organizations involved in delivering, improving access to, or making recommendations for, services for families with children with disabilities. The findings of this inquiry may be applicable to other community-based health care settings. Similar findings have been reported among public health decision-makers in Canada and the US. These studies have reported that public health professionals at all decision-making levels want quick and easy access to synthesized, high-quality evidence that clearly articulates implications for policy and practice [5,27,40]. Given these findings it is likely that health care decision-makers engaged in the provision of health care services to individuals, families, groups, and populations in a variety of community-based settings, experience similar information needs and preferences to the ones reported in this paper. The findings reported in this paper will be particularly useful for health services researchers, especially those in the field of childhood disabilities and research-producing organizations that create research information applicable for use by community-based organizations.

One opportunity available in this study was the possibility to seek the perspectives of people working in three types of organizations whose roles and responsibilities, functions, and relationships with CanChild differed considerably. This afforded the chance to explore both common features across settings and variations by type of organization. It is not surprising that people working in the DHCs, which are research-focused organizations, often expressed different perspectives from practice-based respondents in the CTCs and CCACs concerning their use of research evidence, barriers to use, and organizational culture,. While it is somewhat intuitive that research-generating organizations would report greater use of research evidence in decision-making, it remains unclear if this is the result of research producers being more comfortable with the use of research evidence in general, or if the types of decisions they were engaged in lent themselves more easily to the incorporation of research evidence than those faced by the two more practice-based settings (CTCs and CCACs). Research has shown that the most commonly reported facilitators to the use of research evidence in policy-making are timeliness and relevance of the research, and research that includes a summary with clear recommendations [41,42].

Given that one of the roles of the DHCs was to influence provincial health policies by making recommendations for programs and services, and that DHC's self-reported use of research evidence was fairly high, one could conclude that the research evidence available for the treatment of childhood disabilities was relevant and adequate for the decision-making activities faced by DHCs. While it is not surprising that the DHCs reported higher use of research evidence in their decision-making, fewer barriers to its use, and an organizational culture more conducive to research use, this finding does imply that research-producers like the DHCs can also be important target audiences for research evidence. It would be prudent, therefore, for organizations like CanChild to develop collaborative relationships with organizations like the DHCs, so as to become more familiar with their information needs and preferences, and then develop and implement KT activities to address these needs.

Between the latter two groups of respondents (CTCs and CCACs), there were also some observed differences that likely reflect, among other factors, the more fully established partnership between the CTCs and CanChild. The relationship between the CCACs and CanChild, while established, was still very new. Given their role of coordinating a broad array of services within a complex mandate, the CCACs were not at that time perceived as a key target audience for CanChild's research dissemination program. It might be that greater interaction between researchers at CanChild and the CTCs explains why the CTCs generally reported greater ease of use of recently received research information, higher scores on the extent to which research information was actively sought before making decisions, and fewer perceived barriers to using research evidence in practice than the CCACs. These results are also supported by Innvaer *and colleagues *who found that personal contact between research producers and users was an important facilitator of research use [41].

However, these findings might also reflect that the available research evidence was more relevant and targeted at the type of decisions being made by those in the CTCs than those in the CCACs. For example, some of the Keeping Current pieces disseminated by CanChild focused on the effectiveness of clinical interventions for children with disabilities, as well as the merits of implementing family-centered care. These topics would be applicable not only to decisions clinicians faced in daily practice, but also to managers and directors who might be in the process of improving and revising programs, and to CEOs engaged in broad policy level decisions about service provision generally or in how services could be organized. It is likely that the research evidence disseminated by CanChild at that time was more closely aligned with the types of decisions encountered by the CTCs and therefore encouraged respondents to look for this evidence prior to making practice decisions. Similar findings have been reported by Dobbins and colleagues, who reported that public health decision-makers were significantly more likely to incorporate research evidence into program planning decisions when the evidence was very relevant to the decisions in which they were engaged [39,39]. Others have articulated that an important component of facilitating the use of research evidence is providing evidence to decision-makers that clearly answers their questions [43].

It could be argued, however, that the evidence disseminated by CanChild was more relevant for the CTCs because of the longstanding collaborative relationship that existed between them. By working collaboratively over a number of years, the CTCs were equal partners in identifying research questions that needed to be addressed, and CanChild developed a program of research focused on meeting those needs. More collaborative relationships between research users and producers have been advocated by many as a means of improving research transfer and uptake [3,24,43-46].

Knowledge transfer is a complex phenomenon that includes more than simply getting the right information into the hands of the right people at the right time. It is equally important that health care decision-making be influenced by a combination of clinical judgment, patient preferences, resources, and research evidence [47-49]. However, these findings are noteworthy because they demonstrate the importance of research-producing organizations knowing not only who their target audience(s) are and what their needs are concerning research evidence, but also what questions require answers, and what kind of answers are optimal for different types of decisions. In order to know the needs of their target audiences, researchers and research-producing organizations will have to invest significant effort to identify their target audiences, develop a collaborative relationship, engage meaningfully with them to develop research questions and designs, and work with them to interpret, translate, and apply the results of research evidence into policy and practice. These same messages have been corroborated by others who have advocated for enhanced collaborative relationships between research-producing organizations and intended research users [3,45,50-52].

The results reported in this study illustrate considerable consistency across organization type and position in relation to the preferred methods for receiving research information. There were no significant differences observed by level of decision-maker, and only one difference observed by organization type, with DHCs preferring listservs considerably more than either CTCs or CCACs as a method for receiving research information. The top four methods preferred for receiving research information were websites, health-related research journals, electronic mail, and conferences/workshops. Some of these findings (electronic mail and websites) have been supported by others [5,11], while other research has shown limited preference for conferences/workshops. Generally, studies in this field have indicated that conferences are not an effective way of promoting knowledge transfer and uptake among health service decision-makers, policy-makers and practitioners [20,53,54]. It may be that conferences and workshops needed to be assessed separately in this study, and that participants preferred workshops that were interactive and developed with the needs of users in mind, as opposed to the traditional conference format.

Formats of research information preferred by participants in this study were first, executive summaries, followed by abstracts. The least preferred was full text original articles/reports. These findings continue to highlight that it is important to frame research evidence in ways that are sensitive not only to the needs of various audiences, but also the available resources and skills of those audiences. Similar findings have been reported elsewhere [55-58]. Ely and colleagues suggest that evidence can be provided to primary care physicians at the point of care, but it is most useful when it has been digested into quickly accessible summaries. They further suggest that researchers need to frame their answers to research questions better, and that this would be accomplished by researchers becoming more familiar with the questions that occur in practice/policy-making. In order to become more familiar with the research questions, researchers would have to engage in more meaningful dialogue with target users, which would be facilitated through the development of collaborative relationships [9,44,51].

Cogdill explored the information needs and information-seeking behaviours of nurse practitioners, and found that education or outreach programs can be used to promote the use of information resources to retrieve evidence from clinical research to support various practice decisions [56]. However, these programs must be developed in a way that builds on what is known about the clinician's information needs, as well as how they resolve these needs. For example, a number of studies report that health professionals generally turn to other health professionals first to obtain information to resolve an information need, as opposed to written research reports [55,57,59].

In the study by Thompson and colleagues, it was found that nurses accessed 'evidence-based' information sources in the context of continuing professional development and formal education or training. Other influences included being involved in the production of local protocols and guidelines, interpreting research such as clinical trials, or using research evidence to help resolve conflict between colleagues [57]. The nurses in Thompson's study also articulated what made an information source in this case usually clinical nurse specialists useful: directly answered the question posed; seen to be authoritative and trustworthy, provided or could potentially provide a balance of 'background' (factual) knowledge as well as foreground (management) knowledge; provided supportive and unchallenging information; and had no or minimal associated need for critical appraisal.

Another implication of this observation is the need for researchers and research-producing organizations to 'translate' findings into plain language, devoid of the jargon with which researchers traditionally communicate within the field. Similar findings were reported by Dobbins and colleagues in a study of public health decision-makers [5]. In this national study, public health decision-makers indicated that what they needed most from public health researchers were two-page executive summaries that clearly communicated the issue from a local context, highlighted available evidence, and identified specific practice and policy implications for each evidence point.

An important barrier to implementing the suggestions made in this paper exists for academic researchers. The production of synthesized, relevant, and applied research information that requires the sustainability of collaborative relationships between the researcher and target audiences in order to product these documents, has been less valued for promotion and tenure than peer-reviewed materials. One can only hope that as the imperative of knowledge transfer and exchange becomes more widely valued so too will these translation activities within academic centres. Work is currently ongoing to develop criteria upon which such activities can be included and evaluated for the purposes of tenure and promotion.

The findings of this study provide certain optimism for CanChild in relation to its knowledge transfer and exchange strategy. Generally CanChild is on its way to achieving its goal of promoting evidence-informed decision-making among the CTCs in Ontario. These results also provide direction and guidance to CanChild concerning additional strategies that must be considered and implemented, as well as the identification and development of new collaborative relationships that must be fostered in order to fully realize their mandate.

## Conclusion

The results of this study are useful to health services researchers and research-producing organizations, particularly those involved in producing research information relevant for community-based and childhood disability settings. While there were significant differences between the three types of organizations, there was considerable similarity with respect to the identification of barriers to research transfer as well as the types of activities organizations engage in, either to promote the use of research evidence and/or integrate research findings into program planning. Awareness of these issues will be particularly important for health services researchers in the coming years as pressure to demonstrate the translation of research knowledge into policy and practice becomes more important. The results of this study should provide a starting point upon which researchers could build an interactive, collaborative knowledge transfer strategy, as well as foster more inclusive relationships among researchers and health care decision-makers and professionals.

## Competing interests

The author(s) declare that they have no competing interests.

## Authors' contributions

MD contributed to the conception and design of the study, development of the interview guide, interpretation of the data, and finalizing this manuscript. PR, the primary investigator, contributed to the study conception and design, obtained funding, finalized the data collection tools, oversaw data collection and analysis, and wrote the project report. NP contributed to the study design, development of data collection tools, interpretation of data, and provided feedback on the project report and manuscript drafts. ML contributed to the study conception and design, data interpretation, and provided feedback on the project report and manuscript drafts. AF was responsible for finalizing the data collection tools, participant recruitment, data collection and analysis, and reviewing the project report and manuscript drafts.
